# Children with disrupted attachment histories: Interventions and psychophysiological indices of effects

**DOI:** 10.1186/1753-2000-3-26

**Published:** 2009-09-04

**Authors:** Carlo Schuengel, Mirjam Oosterman, Paula S Sterkenburg

**Affiliations:** 1 Department of Clinical Child and Family Studies, VU University Amsterdam, Van der Boechorststraat 1, 1081 BT Amsterdam, The Netherlands; 2 Department of Psychotherapy, Bartiméus, Doorn, The Netherlands

## Abstract

Diagnosis and treatment of children affected by disruptions of attachment (out of home placement, multiple changes of primary caregiver) is an area of considerable controversy. The possible contribution of psychobiological theories is discussed in three parts. The first part relates the attachment theoretical perspective to major psychobiological theories on the developmental associations of parent-child relationships and emotional response. The second part reviews studies of autonomic reactivity and HPA-axis activity with foster children, showing that foster children show more reactivity within physiological systems facilitating fight or flight behaviours rather than social engagement, especially foster children with atypical attachment behaviour. The third part is focused on treatment of children suffering from the consequences of disrupted attachment, based on a psychotherapy study with psychophysiological outcome measures. Implications are discussed for theory, diagnosis, and intervention.

## Review

In severe and systematic cases of abuse and neglect, out of home care is often used as intervention to stop abuse and neglect. Being placed out of home, children may experience more physical security but not necessarily more emotional security. Young children in particular, who may not yet have had the opportunity to develop secondary attachment relationships, may lose the only source of security and comfort that they had, however fallible or limited it was. Experiments with primates [1,2] and experiments of nature with orphanage children [3,4] suggest that the risks of severe disruptions of attachment are profound. Fortunately, these and other studies also suggest that these risks may be partially offset by a positive and stable caregiving environment. This has led to an interest in the processes that might mediate the impact of attachment disruptions and compensatory attachment experiences on socioemotional development, in particular the pivotal role of attachment relationships with caregivers in the psychophysiological regulation of affect [5-8]. In this contribution, we discuss psychobiological propositions that may complement attachment theory in order to understand the effects of attachment disruptions. Against this background, we discuss studies on children with disrupted attachment in which the quality of their current caregiving experience was related to psychophysiological parameters, using cross-sectional as well as experimental study designs. The findings are discussed with respect to the value of integrating psychobiological theory in the design and evaluation of psychological and behavioural interventions for children with disrupted attachment.

### Psychophysiological regulation of affect and attachment disruptions

Attachment refers to a behavioural propensity to seek contact and proximity to an attachment figure when feeling insecure, due to perceived danger, illness, exhaustion, or other natural cues to danger [9]. In addition, a perceived lack of access to attachment figures is supposed to engender the display of signals of insecurity, which can only be terminated when contact is re-established or an alternative attachment figure is found. Bowlby proposed that responses to separation are the product of the attachment behavioural system, which is organized around the set goal of emotional security. This homeostatic function of the attachment behavioural system, as Bowlby called it, has attracted the attention of psychobiologists such as Hofer [6], who has worked on identifying which neurobiological systems may be involved in organisms' responses to separation or loss.

According to Hofer, parent-child relationships support a range of regulatory processes, including thermoregulation, food intake, tactile stimulation, imitation, and emotional attunement, to name a few. Separation from the parent results in a complete withdrawal of all these regulatory influences, which implicates that children would have to fall back on alternative self-regulatory processes. Because young children's self-regulatory capacities are few and immature, prolonged reliance on these systems may contribute to maladaptive development. Another implication of Hofer's view is that the impact on developmental outcomes of disruptions as well as variations in quality of parent-child interaction may be mediated by multiple and sometimes interrelated regulatory processes, including but not limited to the attachment behavioural system proposed by Bowlby. This would explain why attachment disruptions appear to have consequences for functioning and development that are not confined to the domain of close personal relationships, including behavioural maladjustment and cognitive development [10]. A recent epidemiological study on adult women with a history of foster care reported negative outcomes in the domains of mental and physical health, smoking, educational attainment, obesity, and poverty [11]. Compelling findings in this regard were from a longitudinal sample of high risk families, assessed from infancy to adolescence with a broad array of observational measures and reports from parents as well as teachers [12]. This study showed increases in teacher reported behaviour problems after foster placement, compared to children remaining in their own homes with their maltreating parents, taking into account baseline adaption and SES. In the long term, children placed out of home showed levels of behavioural maladjustment similar to the children remaining with maltreating parents, and heightened as compared to children growing up in high risk families with adequate parenting.

While attachment research is primarily focused on behavioural regulation in parent-child interaction, psychobiological processes are receiving increasing attention. This has been happening in parallel with advances in measuring the output from specific brain and physiological systems that may be related to psychological and behaviour functioning, as well as in parallel with growing theoretical insight in these relations. The current review focuses on the study of psychophysiology and disrupted attachment, and not on cognitive neuroscientific or brain imaging research [13], although this approach is also starting to yield important insight in the development of children with disrupted attachment and the effects of interventions such as foster care [14]. Psychophysiological models provide a further step into advancing our insight into the substrate of behavioural development. Such models have shown to provide a fruitful approach for providing explanations for the developmental links between environmental factors confined to the domain of attachment and outcomes spread across a broad array of other domains, such as mental and even physical health [8]. Psychophysiological models may also be applied in order to expand the range of possible correlates of subjective experiences that may be studied, especially when it is hard for research participants to communicate these experiences, for example because their reports may be biased or because their communicative ability is too low, such as in children or persons with severe learning difficulties. Psychophysiological measures can be taken *in vivo*, which presents opportunities for links with behaviour and social interaction. Psychophysiological research on disrupted attachment is therefore highly relevant from a clinical developmental perspective.

In order to test theoretical predictions using physiological measures, it is necessary to identify which physiological measures are specific indices for the activity of relevant regulatory processes, and under which conditions. Hofer's identification of a multitude of regulatory processes that are hidden in the parent-child relationship warns of an important caveat in deriving from the presence or absence of specific physiological effects the presence or absence of psychological states, such as attachment security. The physiological output from processes supported by parent-child relationships may work in the same or in opposite directions. A separation from the parent may, for example, engender a so-called neuroception of danger and influence arousal in the sympathetic and parasympathetic parts of the autonomic nervous system (ANS) [7]. At the same time, separation from the parent may remove a source of cognitive stimulation, which has also been found to lead to changes in parasympathetic activity [15]. The occurrence of parasympathetic reactivity on separation from a presumed attachment figure therefore leaves open multiple psychological interpretations. Similarly, the absence of discernable physiological reactivity may be equivocal as long as it is possible that another regulatory process has had a counteracting influence on the physiological index.

#### Porges' polyvagal theory

An important step in identifying more specific links between psychophysiology and attachment has been taken by abandoning the concept of general arousal in response to peripheral challenges in favour of an approach that seeks to identify the organization of discrete physiological responses [16]. One avenue for such research has been the polyvagal theory as developed by Porges [17] to describe the neural underpinnings of adaptive and maladaptive autonomic nervous system responses to challenges facing humans. In short, polyvagal theory proposes that human adaptive behaviour is supported by three, hierarchically ordered systems within the autonomic nervous system: the immobilization system, the mobilization system, and the social engagement system.

The *social engagement system *is supposed to be the phylogenetically newest system. It is active when the environment is perceived as safe, and its function is to inhibit bodily processes that interfere with physical growth and restoration (including slowing heart rate, inhibiting fight/flight mechanisms of the sympathetic nervous system, and dampening stress responses across the hypothalamic-pituitary-adrenocortical [HPA]-axis), while facilitating the regulation of muscles, including muscles in the face and head turning muscles used for social engagement behaviour. Neurophysiologically, this system involves activity in the myelinated vagus, implying fast response times and efficient modulation of behavioural responses. This component, which is supposed to originate in the nucleus ambiguous, is therefore nicknamed as the 'smart vagus' [18].

The *mobilization system *is phylogenetically older, and depends on the sympathetic-adrenal system. If harm is perceived as imminent, the influence of the social engagement system 'dissolves' which means that inhibitory influences on heart rate and fight/flight mechanisms are lifted. This gives way to increased cardiac output under the influence of the sympathetic nervous system, decreased expenditure on long-term processes such as growth and immune system activity, and increased expression of the HPA-axis, which are more under the control of limbic structures. This results in a state which might be described as 'arousal', in which psychological states and behaviours such as panic and rage can occur that present a final opportunity for evasive action.

The *immobilization system *would have the oldest phylogenetic roots, and appears to be based in the other, unmyelinated branch of the vagus, also called the vegetative vagus. This system would come into play when the social engagement and the mobilization system both break down. This system facilitates responses that might limit physical damage and increase chances of survival, such as vessel constriction, freezing, and behavioural shutdown. The physiological changes induced can be so massive, that these responses are not without risk to the individual (e.g., when fainting).

The hierarchical organization among these three systems implies a process that links these systems to peripheral systems. Porges proposed a subcortical process called neuroception to monitor the environment for safety, danger, and life threat. Based on recent advances in imaging research, he speculated that neuroception might involve structures in the temporal cortex that evaluate familiar faces, voice, and movements, providing the organism with information about the relative safety of the situation. As a result, for most individuals the presence of familiar persons gives way to activity in the social engagement system, and deactivation of the mobilization and immobilization systems. This allows individuals to affiliate and cooperate, a prominent behavioural feature in our species [19]. Porges [17] proposed that if neuroception goes awry and fails to detect safety in familiar social settings, disordered social behaviour might ensue, such as in autism, social anxiety, post traumatic stress disorder, and reactive attachment disorder. These disorders might therefore be understood as expressions of fight/flight tendencies under conditions that would otherwise allow affiliative and collaborative behaviour, limiting children's opportunities for social and cognitive development, and damaging the social position in the social sphere. Similarly maladaptive may be the failure to activate the mobilization system in risk environments, which appears characteristic of children with William's Syndrome and which may also underlie indiscriminately sociable behaviour towards strangers and straying away of children with Reactive Attachment Disorder, disinhibited type.

In sum, instead of looking merely at general arousal in relation to attachment disruptions, psychobiological research has advanced towards testing physiological indices of specific systems. Polyvagal theory, as an important example, provides a basis for hypothesizing about individual differences in the associations between (experimentally induced) experiences and behavioural states to specific physiological systems [20]. Given the multiple determination of physiological responses, however, caution should be exercised in inferring psychological process from physiological responses [20]. Still, psychobiological mediators of the impact of attachment disruptions on mental and perhaps also physical health [21] are a likely complement to the psychological mediators proposed by Bowlby and Ainsworth in the form of mental models of attachment relationships [13]. For that reason, the success of interventions aimed at children with disrupted attachment may not only be measured at the behavioural and affective-cognitive level, but also at the psychobiological level.

### Psychobiological outcomes of foster care

One of the most invasive interventions in the lives of children is to place them into foster care. As noted, children are not only removed from their familiar surroundings, but they are also separated from their existing attachment figures. For many children placed in foster care, out-of-home placement is the first in a series of placements and disruptions of newly formed attachment relationships with foster parents [22]. These experiences compound the difficulties that these children may already have with stress-regulation, due to the atypical experiences that have led to the foster placement [23]. Psychobiological studies of foster children have focused on ANS reactivity and activity of the HPA-axis.

#### Autonomic reactivity and attachment to foster parents

For foster placement to have a potential beneficial effect on mental health, placements should not only be stable and nurturing, but children must also learn to use their foster parents as external regulators of their psychological and physiological responses to stress. A first study that included measures of ANS reactivity to examine the specific role of foster parents in the regulation system of foster children was reported by Oosterman and Schuengel [24]. ANS reactivity of preschool age foster children (*n *= 60) towards their foster parents was compared to ANS reactivity of children without disruptions in their attachment histories (*n *= 50) towards their parents. Autonomic reactivity was measured during the Strange Situation Procedure [25], using an ambulatory device [26] which continuously recorded heart rate (HR) as a composite measure of general arousal, respiratory sinus arrhythmia (RSA) as a measure of reactivity within the parasympathetic part of the ANS and reflective of activity of the 'smart vagus' [18], and pre-ejection period (PEP) as a measure of reactivity within the sympathetic part of the ANS. Children's responses to separation from their parent or foster parent were reflected in increases in HR and decreases in RSA. This is consistent with studies reporting that RSA decreased after the first separation in the Strange Situation [27,28]. Within Porges' polyvagal theory, the decrease in RSA is interpreted as an indication that the social engagement system was activated by the stressor [17], in this case the separations. There were no significant differences in these responses between foster and control children.

Across the whole Strange Situation Procedure, control children displayed a larger decrease in RSA than foster children, and showed more variability in RSA across the episodes, suggesting that the social engagement system was more active in regulating their responses to separations and reunions with their caregiver as well as the with stranger. Other interpretations are also possible, however. A study with low risk infants with their birth parents found that securely attached infants showed a smaller decrease in RSA during the Strange Situation than children with avoidant attachment, and RSA in securely attached children returned to baseline levels whereas RSA remained significantly below baseline for the avoidant infants [27]. This finding might be taken as a possible contradiction of the view that securely attached infants would be more prone to regulate responses to separations and reunions through social engagement than avoidant attached infants. In order to entangle possible interpretations of differences in physiological responses, it is therefore necessary to study concurrent behavioural indicators and other correlates of RSA. Without directly linking social engagement behaviour to online measures of ANS responses, explanations for the differences in ANS responses throughout the Strange Situation will remain speculative.

A further investigation of the foster group also shed light on correlates of ANS responses in the Strange Situation. It would be expected that foster children as a group, who are likely to have had traumatic experiences and neglect, would not only show less parasympathetic reactivity in the Strange Situation, but would also show more sympathetic reactivity because for these children fight or flight responses might have been more adaptive. However, foster children as a group did not show more sympathetic reactivity within the Strange Situation. Within the group, however, differences were found. Indicators of negative preplacement experiences and disorganized attachment to the foster caregiver were associated with more sympathetic and less parasympathetic reactivity. Increased sympathetic reactivity across the Strange Situation was associated with preplacement experiences of neglect; other indicators of early adversity were not associated with sympathetic reactivity. Within the Strange Situation, quality of attachment was coded for preschool attachment [29]. A distinction was made between ordered types of attachment (secure, avoidant, and ambivalent; 85%) and disorganized types of attachment (disorganized-controlling and insecure-other; 15%). Attachment quality was not associated with preplacement experiences. Children with a disorganized attachment relationship with their foster parent displayed more sympathetic ANS reactivity (PEP decrease) on the first separation as well as across the Strange Situation; they also differed from children with ordered attachment patterns with respect to parasympathetic reactivity, by showing a RSA increase on the first separation, rather than RSA decrease and by showing RSA decrease on the second reunion, rather than RSA increase [30]. These are first findings, requiring replication in other samples of foster children. Other studies that looked at the association between attachment quality and reactivity in the parasympathetic as well as sympathetic part of the ANS across the Strange Situation used another measure of sympathetic reactivity (alpha-amylase [27]), or did not assess disorganized attachment [27,28].

Physiological responses during the Strange Situation also shed an interesting light on behaviour patterns that have been associated with clinically defined disorders of attachment. An interaction effect was found between time in placement and symptoms of disordered attachment, in particular symptoms of indiscriminately friendly/disinhibited behaviour [24]. Based on the idea that attachment relationships need time to develop before they become a source of emotional security, it was expected that children with longer placements would show a stronger increase of RSA (indicative of regulation of the parasympathetic part of the ANS) from separation to reunion with the foster parent in the Strange Situation than children with shorter placements. This was indeed found to be the case, but not for children with symptoms of disinhibited attachment. For the latter group of children, who according to the report by their foster parents showed a clear lack of selective contact seeking when distressed and were prone to wander away with relative strangers, the increase from separation to reunion was weaker when placements had been longer. Symptoms of disordered attachment such as disinhibited behaviour may therefore moderate the outcome of foster placement. In fact, attachment security to the foster parent was only found to be significantly associated with foster parent sensitivity, when symptoms of disordered attachment were taken into account [31]. This pattern of findings indicates that there might be a subgroup of children who might not experience the benefit of a positive caregiving environment, because their interaction with their foster parents is not facilitated by the social engagement system. Instead, there is a subgroup of foster children with disorganized patterns of attachment that appear to resort more to sympathetic reactivity, facilitating fight/flight stress responses.

#### HPA-axis response and intervention programs for foster parents

The HPA-axis has been studied intensively as a system mediating the impact of psychological and physical stressors on behaviour and health. According to Porges [17], the HPA-axis joins the sympathetic part of the ANS within the mobilization system, which becomes active when neuroception of imminent threat occurs, the social engagement system 'dissolves', and the organism has to be prepared for a fight or flight. The HPA-axis expresses itself by secretion of the hormone cortisol, which can be gauged from increased concentrations in blood and saliva. Cortisol facilitates the release of energy but also stimulates the immune system. It acts on receptors in the brain, in a way that is adaptive as a response to acute stress, but might become maladaptive when stress is chronic [32]. Cortisol plays a role maintaining a diurnal pattern, which for humans means that cortisol levels are high after awakening and low at night.

Chronic stress may disrupt the normative diurnal pattern of cortisol, and for this reason has received a fair amount of attention as an indicator of stress and self-regulation among children in foster care. Indeed, it has been found that foster children were more likely than controls to display an abnormally flattened diurnal cortisol pattern, due to very low morning levels or stable high levels [33,34]. In the study of Bruce et al., low morning cortisol was especially associated with a history of severe physical neglect. Based on animal and some human research, low morning cortisol levels may be the result of exposure to chronic stress resulting from abuse or neglect [35]. But not all foster children may have had a similar experience. Bruce and her colleagues also identified foster children who had primarily been exposed to emotional abuse. These children showed atypical high levels of morning cortisol. This may be explained by the more acute, episodic stress associated with emotional abuse [36]. These findings underline the need for more research to tease out whether patterns of physiological functioning of foster children are due to the out of home placement (as a disruption of attachment) or to aberrant experiences that precede out of home placement. In both cases, however, foster children are to be considered a group at risk for atypical psychophysiological development.

Fortunately, however, in independent investigations, Dozier and her colleagues as well as Fisher and his colleagues have provided experimental evidence suggesting that cortisol patterns may be changed towards more adaptive patterns using interventions with foster parents. Dozier developed the Attachment and Biobehavioral Catch-up (ABC) program for new foster parents through the first months of placement, to help them in 4 to 5 home visits to promote secure attachment through nurturing care and in 5 to 6 home visits to regulate behaviour and physiology. The randomized trial showed heightened cortisol levels in a lab setting among the foster children in the control treatment compared to foster children in the ABC-condition [37].

Fisher and his colleagues evaluated the effect of Multidimensional Treatment Foster Care for preschoolers (MTFC-P), an intensive therapeutic foster care program for high risk foster children [38]. This program involves intensive training for foster parents in behaviour management, support from a multidisciplinary team throughout placement, telephone-based monitoring and guidance of foster parents, and therapeutic play group activities for the children. It was found that over time, children in the intervention group maintained a diurnal cortisol pattern more typical of their comparison-group of nonmaltreated children with low-income families, whereas the diurnal pattern in children in the regular foster care condition increasingly became abnormally flattened. Fisher and Stoolmiller [23] showed that these effects appeared to be partially mediated by the reduction of stress in foster caregivers within the MTFC-P condition.

Together, the first psychophysiological studies on foster care shed more light on the role that foster parents may play in changing the developmental pathways of neglected and maltreated children. Foster parents are not only important for supporting behavioural regulation but also for affect regulation, an important principle that can be traced back to the observations that James and Joyce Robertson made on the basis of their films of the children they fostered during short separations from their parents [39]. Symptoms of disinhibited attachment have to be taken serious as possible signs that separations and reunions with foster parents may indeed have a qualitatively different meaning for some foster children, perhaps because the relationship with foster parents plays a less central role in the regulation of affect. This possible interpretation is consistent with Porges' idea that children with attachment disorder are more prone to activate the mobilization system than to activate their social engagement system to deal with challenges under conditions of relative safety [17]. However, interventions to support foster parents, even foster parents of hard-to-place children, show promise for improving psychophysiological regulation in children. This may help to prevent negative outcomes of foster care along a broad array of domains [12].

### Psychophysiological outcomes of psychotherapy

Some children with a history of severe disruption or deprivation of attachment develop disorders of attachment. Several descriptions and criteria for disorders of attachment exist for young children, but consensus is emerging that there is a small group of children who fail to develop specific attachment relationships or show behaviours antithetical to maintaining specific attachments, due to the pathogenic environment in which they grow up [4,40-44]. The most well known distinction is between subtypes of reactive attachment disorder, also called disorders of nonattachment [41]. One subtype describes children with emotionally withdrawn behaviour who strongly inhibit attachment behaviour in situations which are expected to elicit seeking or accepting contact with a familiar caregiver. The other subtype describes children with indiscriminate proximity seeking to familiar or unfamiliar persons, showing a disinhibition of contact seeking behaviour. Although DSM-IV describes these subtypes as distinct and mutually exclusive, considerable overlap in symptoms of these subtypes has been found among institutionalized Romanian children [45]. Disinhibited attachment was found in a substantial minority of children adopted in the UK out of Romanian orphanages, was associated with severe mental health problems, and showed strong persistence within the period between 6 and 11 years of age [4]. Less is known about the course of the inhibited attachment subtype. These disorders of nonattachment are to be distinguished from forms of insecure or disorganized attachment, as well as disorder within specific attachment relationships (attachment disorder with self-endangering, with inhibition, or with compulsive compliance [41]) or temporary reactions to disruptions of attachment [42].

In addition to the plethora of possible forms in which attachment may be disordered, considerable debate exists about the appropriate therapeutic response for these children. Based on the available theoretical and empirical evidence, as well as clinical consensus, Boris and Zeanah outlined recommendations [40]. After ascertaining safety for the child, the preferred avenue for treatment is working with the children's parents or regular caregivers, based on methods that have been proven effective in promoting security of attachment among parents and children at risk for insecure attachment [46]. Interventions may be said to be effective for children with disordered attachment, if children show that they become able to use the prospective attachment figure as a source of comfort and a secure base for exploration and learning. In Hofer's [6] terms, the intervention should establish or re-establish regulatory processes embedded within child-caregiver relationships, and in particular regulation of emotional distress. In terms of Porges' polyvagal theory, intervention is successful when children become more able to use the social engagement system to deal with challenges in the presence of a familiar caregiver or attachment figure. In order to test these outcomes, psychophysiological measures may be an important complement to behaviour observation, in order to provide a window to changes in emotion regulation and the social engagement system.

Although psychophysiological measures are still being used sparsely in measuring changes in emotions during psychotherapy [47], successful examples do exist where cortisol sampling and cardiography are used to obtain objective indicators of treatment outcome (because these data are provided 'blind' to the treatment condition) as well as insight into the psychotherapeutical process [48-50]. We used psychophysiological measures to test the effects of a psychotherapeutical approach that was specifically designed for non-autistic children with disrupted attachment histories, severe behaviour problems, and moderate to severe intellectual and visual disabilities [51-53].

There are a small group of children who grow up with a combination of serious risk factors as well as severe learning difficulties. Due to intensive care needs or vulnerability in the family, not all families are able to adequately care for their child themselves. Unfortunately, these children are often also difficult to place in foster families. As a result, some of these children grow up in group homes. Not only do these children experience instability of placement, but in group homes, they are confronted with multiple caregivers. Due to their intellectual limitations, it may be difficult for these children to develop selective attachment relationships with these caregivers. Left to their own, underdeveloped skills for dealing with stress, maladaptive forms of coping may develop. This may be one of the reasons why aggression to self, to others, as well as extremely withdrawn behaviour may be so heightened among children with intellectual disabilities [54]. The problem is that these challenging behaviours require a high level of caution, tolerance, and experience on the part of caregivers who may attempt to connect to these children in a way which may develop into an attachment relationship. For these reasons, a treatment protocol was developed, called Integrative Therapy for Attachment and Behavior (ITAB) [53].

The first phase of treatment is that an experienced and trained psychotherapist attempts to build an attachment relationship with the child. The therapist makes verbal and tactile contact and invites the client to engage in interaction. The interactions typically involve imitation and play. The therapist is sensitive to signs of resistance or discomfort, and never coerces the child into contact. Gradually the therapists attempts to extend the chains of mutual responsiveness, introduces variations and games, and uses soothing responses to facilitate the regulation of the client's affective reactions, which may occur on reunion or at leave-taking, but which may also occur when the joint activities generate affective arousal. The therapist will be watching for evidence of object permanence and person permanence, because this is important for building up a representation of the therapist. As soon as the therapist perceives evidence to this effect (searching, recognition), the therapist will build on this by introducing games and activities (peek-a-boo) in which person permanence is further trained. The therapist will also stimulate and reward the expression of wishes and desires, in order to strengthen the representation of a responsive attachment figure. The first phase consists of three sessions a week, across a period of six months or longer in order for the client to learn about the therapist and recognize her, and build up expectations about her responsiveness. After this phase of building a therapeutic attachment relationship, the second phase uses this relationship as a secure context in which the child might be able to learn new adaptive behaviour to replace the maladaptive problem behaviour. This is done by analyzing the conditions which continue to give rise to challenging behaviour, defining appropriate replacement behaviours, and using positive social reinforcement for training these behaviours. Within the final phase, the psychotherapist works with the regular caregivers to expand the attachment network of the child. Gradually, the relationship with the psychotherapist moves to the background.

A controlled multiple case study was conducted with six children between age 10 and 17 years old with a long history of disrupted attachment and IQs between 20 and 35 Clients were blind or visually impaired according to WHO criteria. Clients were assigned for this intensive form of psychotherapy if a documented history showed extensive disruptions of attachment (early neglect, early institutional placement, placement shifts), challenging behaviour was severe, medical causes for the behaviour were ruled out, and behavioural interventions with the caregivers or others had failed. Most children lived quite isolated lives in their group home, because the professional caregivers were hesitant to remain in the vicinity. Only one child received part-time care from his adoptive parents. The first question was whether these children would begin to seek proximity to the therapist in times of stress. A second question was whether having to learn new behaviour in challenging situations would be more successful with ITAB, as compared to regular behaviour modification, and whether clients would show less stress reactivity, in particular in the mobilization system. Sessions with the experimental ITAB therapist alternated with a session with a control therapist on the same day. During phase 1, the control therapist was only positively involved towards the client but did not take the initiative to build a relationship. The length of phase 1 was for the study manipulated to vary, in order to create a multiple baseline design. During phase 2, this therapist applied the same behaviour modification protocol as the ITAB therapist for learning new, more adaptive behaviour in the challenging situation. Each session was videotaped, and coded in random order by trained observers blind to phase and condition. During the sessions, ambulatory recording was done of PEP, to measure reactivity in the sympathetic part of the ANS and an index of the mobilization system, and RSA, a parasympathetic indicator and reflective of the social engagement system. Appropriate controls were used for artefacts due to locomotion [51].

Using the RSA and PEP data, we identified, within each of the sessions within phase 1, episodes of heightened sympathetic or parasympathetic arousal and coded the duration and frequency of proximity seeking to the therapist (ITAB or control). A significant difference was found, meaning that proximity seeking to the ITAB therapist increased during periods of heightened arousal, whereas it remained stably low for the control therapist. This indicated that even after a long history of deprivation, children may cease to inhibit their attachment behaviour and may start approaching responsive people in their environment in times of stress. However, these findings are inconclusive, because stress was not experimentally induced [20], which meant that the peak periods of ANS reactivity may have been caused by stress but also by attention, excitement, or even metabolic processes.

In the second phase of psychotherapy, however, situations were identified which were presumably challenging or frustrating because they elicited problem behaviour (such as biting, scratching, spitting). Session with the ITAB therapist were expected to provide a better context for learning alternative, adaptive behaviours to replace these problem behaviours, than sessions with the control therapist, because these children might not perceive the situation as safe with the control therapist. Within Porges' [17] model, this would lead to reactivity in the mobilization system. Indeed we found that children learned to use adaptive behaviours more frequently in the sessions with the ITAB therapist [53]. Furthermore, we found that in four out of the six children, the therapeutic relationship with the ITAB therapist prevented the activation of the mobilization system, because sympathetic reactivity increased during behaviour modification with the control therapist but remained stably low during behaviour modification with the ITAB therapist [51]. These findings suggest that for clients who before did not approach caregivers and appeared indifferent to human contact, the interaction with a specific person had become a support for the regulation of physiological reactivity in challenging situations.

## Conclusion

Psychobiological theory may be an important complement to theories such as attachment theory to not only explain the consequences of the complex experiences surrounding disruptions of attachment, but also to identify mechanisms through which developmental change can be effected. The relationship between children and foster parents may contain the 'hidden regulators' that Hofer [6] proposed as typical for regular attachment relationships, as shown by the parasympathetic nervous system responses to separations and reunions [24] and by the effects of interventions aimed towards improving the interaction between children and foster parents or therapeutic workers [37,38,51] on physiological indices of affect regulation. Relationships between children with disrupted attachment and their caregivers are therefore an important focus for research on their developmental pathways, as well as a focus for support and intervention in order to promote more adaptive developmental outcomes.

For some children, however, the regulating function of their relationships with caregivers appears compromised. Within Porges' theory, this would be the case when situations that would be safe enough for social engagement to be activated, elicit responses that facilitate mobilization of resources for fight or flight [7,17]. In his theory, processes on a subcortical level (e.g., the limbic system) operate without cognitive awareness in order to distinguish between situations that are safe, dangerous, or life threatening. Mechanisms that facilitate recognition of visual and auditory patterns are probably involved in this so called neuroception of safety and danger [17]. Little is known about the kinds of experiences that may lead to aberrant neuroception. Neuroception of danger would predispose to reactivity of the sympathetic part of the ANS, called the mobilization system in polyvagal theory, and would weaken parasympathetic reactivity or vagal tone. The link found between sympathetic ANS reactivity and a history of neglect suggests one avenue for further research. Another relevant finding was the low parasympathetic reactivity during reunion with foster parents by children showing symptoms of disinhibited disordered attachment [24]. Clinically, children with disorders of attachment appear to have pervasive disturbances in social relatedness and in particular using familiar caregivers as a source of comfort and safety [40], and these disturbances appear to be persistent [4]. Distortions in neuroception of safety and danger may be one explanation for this persistence, and therefore a potential target for intervention. Links between disorganized attachment relationships and sympathetic reactivity on reunion with the caregiver would in Porges' framework be highly consistent with the interpretation by attachment theorists that disorganized attachment patterns are the result of the opposing tendencies engendered by the fear system (fight or flight) and the attachment system (seek contact) [55]. These direct links have, however, not been found in the one study that examined this. An further important test of Porges' theory as well as the theories regarding disorganized attachment would therefore be to investigate the extent to which sympathetic ANS reactivity on reunion with caregivers mediates the link between frightening experiences with these caregivers and disorganized attachment behaviour. Furthermore, Porges' propositions that neuroception is based on systems for feature and movement detection, localized within the temporal cortex [17] require the extension of psychophysiological measures with imaging techniques [13].

Psychobiological perspective and research findings may also contribute to rational interventions for children with disrupted attachment histories. These interventions should not only be effective, but also safe (unlike coercive treatments that are labelled by their proponents as 'attachment therapies' [56]). If support within children's own families is not effective or feasible and children have to be placed out of home, the developmental risks of foster care show that more is needed than a physically safe family. Emerging findings have been reviewed which suggested that well-designed interventions aimed at foster parents may nudge back psychophysiological parameters within the normative range. Even for children with intellectual disabilities, the interactions between behaviour, environment, and psychophysiology appeared malleable by psychotherapy (ITAB) consisting of sensitivity and taking the time to get to know the child and to allow the child to get to know the therapist. More research is needed to test whether specific activities within the ITAB protocol, such as games with disappearing and returning and making and breaking contact, might derive their effect by stimulating the experience of familiarity and recognition, stimulating neuroception of safety in Porges' terms.

In any case, psychophysiological measures prove useful because they demonstrate that intervention not only change outward behaviour, but hidden physiological responses change as well. Rather than giving up on the potential of children severely affected by disruptions of attachment to participate in mutually fulfilling social relationships, practice may build on the initial findings reviewed to expand the number of evidence based interventions on offer for this vulnerable group of children.

## Competing interests

The authors declare that they have no competing interests.

## Authors' contributions

CS conceived of the review topic and outline, and drafted the manuscript. MO and PSS conducted the research which formed a major part of this review, and helped to draft the manuscript. All authors read and approved the final manuscript.

## References

[B1] Van IJzendoorn MH, Bard KA, Bakermans-Kranenburg MJ, Ivan K (2009). Enhancement of attachment and cognitive development of young nursery-reared chimpanzees in responsive versus standard care. Developmental Psychobiology.

[B2] Harlow H (1958). The nature of love. American Psychologist.

[B3] Nelson CA, Zeanah CH, Fox NA, Marshall PJ, Smyke AT, Guthrie D (2007). Cognitive recovery in socially deprived young children: The Bucharest early intervention project. Science.

[B4] Rutter M, Colvert E, Kreppner J, Beckett C, Castle J, Groothues C, Hawkins A, O'Connor TG, Stevens SE, Sonuga-Barke EJ (2007). Early adolescent outcomes for institutionally-deprived and non-deprived adoptees. I: Disinhibited attachment. Journal of Child Psychology and Psychiatry.

[B5] Bradley SJ (2000). Affect regulation and the development of psychopathology.

[B6] Hofer MA (2006). Psychobiological roots of early attachment. Current Directions in Psychological Science.

[B7] Porges SW (2003). Social engagement and attachment: A phylogenetic perspective. Annals of the New York Academy of Sciences.

[B8] Schore AN (2001). Effects of a secure attachment relationship on right brain development, affect regulation, and infant mental health. Infant Mental Health Journal.

[B9] Bowlby J (1984). Attachment and loss: Attachment.

[B10] Rutter M, O'Connor TG, English and Romanian Adoptees (ERA) Study Team (2004). Are there biological programming effects for psychological development?: Findings from a study of Romanian adoptees. Developmental Psychology.

[B11] Schneider R, Baumrind N, Pavao J, Stockdale G, Castelli P, Goodman GS, Kimerling R (2009). What happens to youth removed from parental care?: Health and economic outcomes for women with a history of out-of-home placement. Children and Youth Services Review.

[B12] Lawrence CR, Carlson EA, Egeland B (2006). The impact of foster care on development. Development and Psychopathology.

[B13] Fox NA, Hane AA, Cassidy J, Shaver PR (2008). Studying the biology of human attachment. Handbook of attachment: Theory, research, and clinical applications.

[B14] Marshall PJ, Reeb BC, Fox NA, Nelson CA, Zeanah CH (2008). Effects of early intervention on EEG power and coherence in previously institutionalized children in Romania. Development and Psychopathology.

[B15] Richards JE, Casey BJ (1991). Heart-rate-variability during attention phases in young infants. Psychophysiology.

[B16] Fox NA, Card JA, Cassidy J, Shaver PR (1999). Psychophysiological measures in the study of attachment. Handbook of attachment: Theory, research, and clinical applications.

[B17] Porges SW (2007). The polyvagal perspective. Biological Psychology.

[B18] Beauchaine TP, Gatzke-Kopp L, Mead HK (2007). Polyvagal Theory and developmental psychopathology: Emotion dysregulation and conduct problems from preschool to adolescence. Biological Psychology.

[B19] Flinn MV, Geary DC, Ward CV (2005). Ecological dominance, social competition, and coalitionary arms races: Why humans evolved extraordinary intelligence. Evolution and Human Behavior.

[B20] Berntson GG, Cacioppo JT, Grossman P (2007). Whither vagal tone. Biological Psychology.

[B21] Meredith P, Ownsworth T, Strong J (2008). A review of the evidence linking adult attachment theory and chronic pain: Presenting a conceptual model. Clinical Psychology Review.

[B22] Oosterman M, Schuengel C, Slot NW, Bullens RAR, Doreleijers TAH (2007). Disruptions in foster care: A review and meta-analysis. Children and Youth Services Review.

[B23] Fisher PA, Stoolmiller M (2008). Intervention effects on foster parent stress: Associations with child cortisol levels. Development and Psychopathology.

[B24] Oosterman M, Schuengel C (2007). Autonomic reactivity of children to separation and reunion with foster parents. J Am Acad Child Adolesc Psychiatry.

[B25] Ainsworth MDS, Wittig BA, Foss BM (1969). Attachment and exploratory behavior of one year olds in a strange situation. Determinants of infant behavior.

[B26] De Geus EJC, Van Doornen LJP, Fahrenberg J, Myrtek M (1996). Ambulatory assessment of parasympathetic/sympathetic balance by impedance cardiography. Ambulatory assessment: Computer assisted psychological and psychophysiological methods in monitoring and field studies.

[B27] Hill-Soderlund AL, Mills-Koonce WR, Propper C, Calkins SD, Granger DA, Moore GA, Gariepy JL, Cox MJ (2008). Parasympathetic and sympathetic responses to the strange situation in infants and mothers from avoidant and securely attached dyads. Developmental Psychobiology.

[B28] Oosterman M, Schuengel C (2007). Physiological effects of separation and reunion in relation to attachment and temperament in young children. Developmental Psychobiology.

[B29] Cassidy J, Marvin RS, John D, Catherine T (1992). Attachment organization in preschool children: Procedures and coding manual Seattle, WA.

[B30] Oosterman M, De Schipper JC, Fisher PA, Dozier M, Schuengel C Autonomic reactivity in relation to attachment and early adversity among foster children. Development and Psychopathology.

[B31] Oosterman M, Schuengel C (2008). Attachment in foster children associated with caregivers' sensitivity and behavioral problems. Infant Mental Health Journal.

[B32] Fisher PA, Gunnar MR, Dozier M, Bruce J, Pears KC (2006). Effects of therapeutic interventions for foster children on behavioral problems, caregiver attachment, and stress regulatory neural systems. Resilience in Children.

[B33] Dozier M, Peloso E, Lindhiem O, Gordon MK, Manni M, Sepulveda S (2006). Developing evidence-based interventions for foster children: An example of a randomized clinical trial with infants and toddlers. Journal of Social Issues.

[B34] Bruce J, Fisher PA, Pears KC, LeVine S (2009). Morning cortisol levels in preschool-aged foster children: Differential effects of maltreatment type. Developmental Psychobiology.

[B35] Sanchez MM, Ladd CO, Plotsky PM (2001). Early adverse experience as a developmental risk factor for later psychopathology: Evidence from rodent and primate models. Development and Psychopathology.

[B36] Kaufman J, Birmaher B, Perel J, Dahl RE, Moreci P, Nelson B, Wells W, Ryan ND (1997). The corticotropin-releasing hormone challenge in depressed abused, depressed nonabused, and normal control children. Biological Psychiatry.

[B37] Dozier M, Peloso E, Lewis E, Laurenceau JP, LeVine S (2008). Effects of an attachment-based intervention on the cortisol production of infants and toddlers in foster care. Development and Psychopathology.

[B38] Fisher PA, Stoolmiller M, Gunnar MR, Burraston BO (2007). Effects of a therapeutic intervention for foster preschoolers on diurnal cortisol activity. Psychoneuroendocrinology.

[B39] Robertson J, Robertson J (1971). Young children in brief separation: A fresh look. Psychoanalytic Study of the Child.

[B40] Boris NW, Zeanah CH, Work Group on Quality Issues (2005). Practice parameter for the assessment and treatment of children and adolescents with reactive attachment disorder of infancy and early childhood. Journal of the American Academy of Child and Adolescent Psychiatry.

[B41] Boris NW, Hinshaw-Fuselier SS, Smyke AT, Scheeringa MS, Heller SS, Zeanah CH (2004). Comparing criteria for attachment disorders: Establishing reliability and validity in high-risk samples. Journal of the American Academy of Child and Adolescent Psychiatry.

[B42] Zilberstein K (2006). Clarifying core characteristics of attachment disorders: A review of current research and theory. American Journal of Orthopsychiatry.

[B43] O'Connor TG, Zeanah CH (2003). Attachment disorders: Assessment strategies and treatment approaches. Attachment and Human Development.

[B44] DeKlyen M, Greenberg MT, Cassidy J, Shaver PR (2008). Attachment and psychopathology in childhood. Handbook of attachment: Theory, research, and clinical applications.

[B45] Zeanah CH, Smyke AT, Koga SF, Carlson E (2005). Attachment in institutionalized and community children in Romania. Child Development.

[B46] Bakermans-Kranenburg MJ, Van IJzendoorn MH, Juffer F (2003). Less is more: Meta-analyses of sensitivity and attachment interventions in early childhood. Psychological Bulletin.

[B47] Sloan DM, Kring AM (2007). Measuring changes in emotion during psychotherapy: Conceptual and methodological issues. Clinical Psychology-Science and Practice.

[B48] Griffin MG, Nishith P, Resick PA, Yehuda R (1997). Integrating objective indicators of treatment outcome in posttraumatic stress disorder. Psychobiology of Posttraumatic Stress Disorder.

[B49] Lindauer RTL, van Meijel EPM, Jalink M, Olff M, Carlier IVE, Gersons BPR (2006). Heart rate responsivity to script-driven imagery in posttraumatic stress disorder: Specificity of response and effects of psychotherapy. Psychosomatic Medicine.

[B50] Olff M, de Vries GJ, Guzelcan Y, Assies J, Gersons BPR (2007). Changes in cortisol and DHEA plasma levels after psychotherapy for PTSD. Psychoneuroendocrinology.

[B51] Schuengel C, Sterkenburg PS, Jeczynski P, Janssen CGC, Jongbloed G (2009). Supporting affect regulation in children with multiple disabilities during psychotherapy: A multiple case design study of therapeutic attachment. Journal of Consulting and Clinical Psychology.

[B52] Sterkenburg P, Schuengel C, Janssen C (2008). Developing a therapeutic relationship with a blind client with a severe intellectual disability and persistent challenging behaviour. Disability and Rehabilitation.

[B53] Sterkenburg PS, Janssen CGC, Schuengel C (2008). The effect of an attachment-based behaviour therapy for children with visual and severe intellectual disabilities. Journal of Applied Research in Intellectual Disabilities.

[B54] Schuengel C, Janssen CGC (2006). People with mental retardation and psychopathology: Stress, affect regulation and attachment. A review. International Review Of Research In Mental Retardation.

[B55] Main M, Hesse E, Greenberg MT, Cicchetti D, Cummings EM (1990). Parents' unresolved traumatic experiences are related to infant disorganized attachment status: Is frightened and/or frightening parental behavior the linking mechanism?. Attachment in the preschool years: Theory, research, and intervention.

[B56] Chaffin M, Hanson R, Saunders BE, Nichols T, Barnett D, Zeanah C, Berliner L, Egeland B, Newman E, Lyon T, LeTourneau E, Miller-Perrin C (2006). Report of the APSAC task force on attachment therapy, reactive attachment disorder, and attachment problems. Child Maltreatment.

